# Frequency-reduction strategy of roxadustat in patients undergoing peritoneal dialysis: a multi-center retrospective cohort study

**DOI:** 10.3389/fmed.2025.1708916

**Published:** 2026-01-14

**Authors:** Xi Mi, Guanying Lin, Yijing Fang, Lu Zhu, Yanhong Lin, Tingting Zhang, Xiaohong Zhong, Xiaoyan Su, Xianrui Dou, Jun Ai

**Affiliations:** 1 Division of Nephrology, Nanfang Hospital, Southern Medical University, Guangzhou, China; 2 National Clinical Research Center for Kidney Disease, Guangzhou, China; 3 State Key Laboratory of Organ Failure Research, Guangzhou, China; 4 Guangdong Provincial Institute of Nephrology, Guangzhou, China; 5 Guangdong Provincial Key Laboratory of Renal Failure Research, Guangzhou, China; 6 Department of Orthopaedics and Traumatology, Nanfang Hospital, Southern Medical University, Guangzhou, China; 7 Department of Nephrology, Dongguan Tungwah Hospital, Dongguan, China; 8 Dongguan Key Laboratory of Precise Prevention & Treatment of Chronic Kidney Disease and Complications, Dongguan, China; 9 The Eighth Affiliated Hospital, Southern Medical University (The First People’s Hospital of Shunde), Foshan, Guangdong, China

**Keywords:** dose reduction, frequency reduction, hemoglobin stability, peritoneal dialysis, roxadustat tapering strategies

## Abstract

**Background:**

Although dose reduction is the guideline-recommended tapering strategy for roxadustat to maintain target hemoglobin levels in peritoneal dialysis (PD) patients during the maintenance phase, reducing the administration frequency represents another potential clinical approach. However, the specific efficacy of this strategy in sustaining target hemoglobin levels remains unclear.

**Methods:**

This was a retrospective cohort study conducted from 1 January 2021 to 31 December 2024, enrolling PD patients from three dialysis centers in South China. Participants who achieved target hemoglobin levels were stratified into two groups based on roxadustat tapering strategies: The dose-reduction group, in which the per-dose amount was gradually decreased while maintaining the administration frequency, and the frequency-reduction group, in which the administration frequency was reduced without changing the per-dose amount. During a 12-month follow-up period, with assessments conducted every 3 months, mean hemoglobin levels, hemoglobin target attainment rates, and hemoglobin variability—evaluated using the residual standard deviation (RSD) method—were compared between the two tapering strategies. The association between hemoglobin target non-attainment and tapering strategies was analyzed using Cox proportional hazards models.

**Results:**

Among the 402 PD patients included in the analysis, the mean age was 45.3 ± 13.8 years and 55.5% were male. No significant difference was observed in the hemoglobin change trend and mean hemoglobin levels between the two groups throughout the follow-up period. Compared to the dose-reduction group, the patients who reduced dosing frequency demonstrated significantly higher hemoglobin target attainment rates at months 3 (64.1% vs. 40.4%), 6 (55.6% vs. 43.2%), and 12 (49.2% vs. 36.6%; all *p* < 0.05). In addition, the patients in this group exhibited a lower mean Res-SD value (12.3 vs. 15.5; *p* < 0.05). Moreover, the frequency-reduction strategy was associated with a significantly lower risk of hemoglobin target non-attainment (adjusted hazards ratio [HR] 0.64, 95% CI 0.50–0.82; *p* < 0.001).

**Conclusion:**

Compared to the dose-reduction group, the frequency-reduction group showed higher hemoglobin target attainment rates, lower hemoglobin variability, and a reduced risk of hemoglobin target non-attainment. The frequency-reduction strategy appears to be a potential tapering approach for peritoneal dialysis (PD) patients.

## Introduction

Chronic kidney disease (CKD) is one of the significant contributors to the global health burden, affecting more than 10% of the world’s population (1). Renal anemia, a common complication of CKD, has an incidence exceeding 50% in patients with Stage 5 CKD (2), with prevalence rates surpassing 50% in non-dialysis and 90% in dialysis-dependent populations (3). Evidence indicates that renal anemia not only impairs quality of life but also accelerates CKD progression (4–6). Despite treatment with iron supplementation and erythropoiesis-stimulating agents (ESAs), a significant proportion of patients with CKD struggle to maintain hemoglobin levels within the KDIGO guideline-recommended target range (110–130 g/L) (7). Even after temporary anemia correction, hemoglobin levels in these patients often show greater variability—fluctuating more widely around the target range compared to healthy individuals (8). Such variability has been linked to increased risks of hospitalization, cardiovascular events, and mortality in some reports (9–12). These fluctuations are attributable to blood loss during hemodialysis, inflammatory conditions, endogenous erythropoietin (EPO) resistance, and modifications in the dosing of exogenous erythropoietic agents.

In this context, roxadustat has emerged as a groundbreaking therapeutic alternative for renal anemia. As an oral hypoxia-inducible factor prolyl hydroxylase inhibitor (HIF-PHI), roxadustat mimics the body’s physiological response to hypoxia, thereby enhancing endogenous erythropoietin (EPO) production and improving iron metabolism (13). Clinically, roxadustat has been proven to remain equally effective in patients with EPO resistance or underlying inflammatory conditions (14). Notably, recent evidence suggests that roxadustat induces lower hemoglobin variability compared to erythropoiesis-stimulating agents (ESAs) in hemodialysis patients (15).

Since its approval, roxadustat dosage optimization has been a clinical priority. Prior studies have extensively explored optimal initial strategies—including dose and frequency—to achieve hemoglobin correction in both dialysis-dependent and non-dialysis-dependent CKD populations (16–20). However, only a few studies have been conducted on tapering strategies during maintenance therapy. Current guidelines predominantly recommend dose reduction as the exclusive tapering strategy after achieving hemoglobin targets (21). Given that both dose magnitude and administration frequency are adjustable during clinical therapy (19, 20), frequency reduction might be a potential tapering strategy during the maintenance phase. To explore this possibility, we conducted a multi-center retrospective cohort study comparing dose-reduction and frequency-reduction strategies in terms of longitudinal hemoglobin control among peritoneal dialysis (PD) patients who had achieved target levels.

## Methods

### Study population

**Figure 1:**
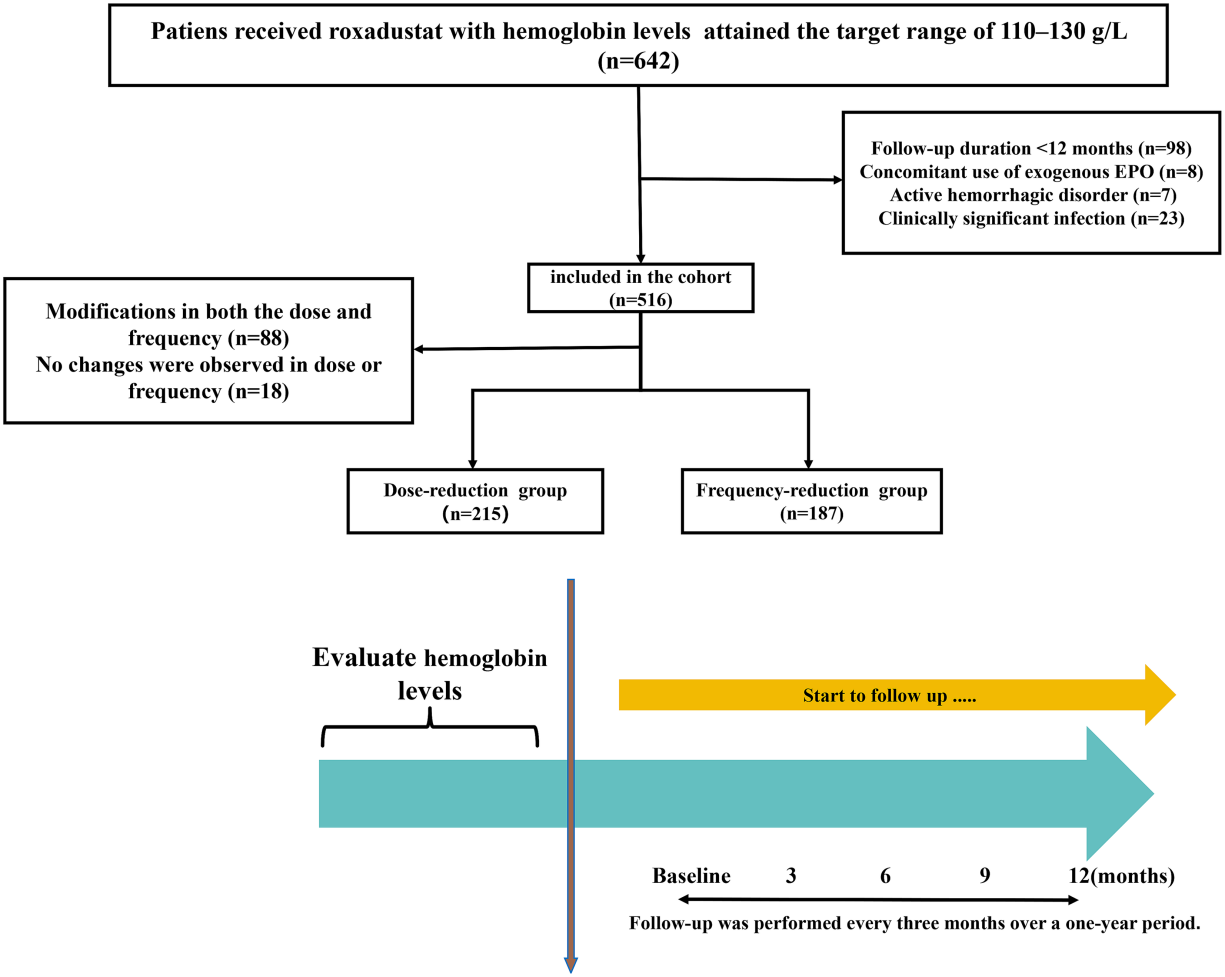
Flowchart of patient selection into the study cohort. EPO, erythropoietin.

This multi-center retrospective cohort study was conducted across three dialysis centers in Southern China—Nanfang Hospital, the First People’s Hospital of Foshan, and Shunde Hospital of Southern Medical University—from 1 January 2021 to 31 December 2024. The initial cohort comprised 642 adult patients on maintenance peritoneal dialysis (PD) who had received roxadustat for ≥ 4 weeks and achieved target hemoglobin levels (110–130 g/L). After applying the exclusion criteria, 227 patients were excluded for the following reasons: (i) follow-up duration less than 12 months (98), (ii) concomitant use of exogenous erythropoietin (8), (iii) active hemorrhagic disorder (7), and (iv) clinically significant infection (23). Among the 516 eligible participants, 88 patients underwent adjustments in both roxadustat dose and frequency, while 18 maintained unchanged regimens. Consequently, 402 patients were included in the final cohort analysis (Figure 1).

### Inclusion criteria

1. Patients aged > 18 years.
2. CKD patients on PD for more than 3 months.
3. Patients diagnosed with renal anemia and treated with roxadustat for ≥ 4 weeks.
4. Patients with hemoglobin levels within the range of 110–130 g/L (inclusive).
5. Patients with body weight between 45 and 100 kg (inclusive).

### Exclusion criteria

1. Evidence of any clinically significant infection.
2. Concomitant use of exogenous erythropoietin.
3. History of malignancy.
4. Active hemorrhagic disorder.
5. Anemia due to causes other than chronic kidney disease.
6. Blood transfusion within 12 weeks prior to baseline.
7. Pregnancy.
8. Life expectancy less than 12 months.

### Data collection and measurement

Baseline demographic characteristics (age, sex, and body mass index [BMI]) were collected from medical records. Systolic blood pressure (SBP), diastolic blood pressure (DBP), laboratory parameters (including blood hemoglobin, serum creatinine, serum albumin, total cholesterol, C-reactive protein, and serum ferritin), and PD-related characteristics (dialysate glucose concentration [GLUC], 24-h ultrafiltration volume, 24-h urine volume, and weekly total Kt/V) were collected at baseline and at each follow-up visit. All biochemical parameters were measured using standardized, automated methods.

Conventional weekly total Kt/V was measured using standard methods (22).


$$\begin{array}{l} \text{Dialysate glucose concentration}\;(\%)\\ =\sum (\text{glucose concentration}\times \text{input volume})\\ /\text{total input volume} \end{array}$$

The hemoglobin target achievement rate was defined as the percentage of patients whose hemoglobin levels were maintained within the target range of 110–130 g/L at every measurement, in accordance with the KDIGO guidelines.

A patient was considered to have achieved the hemoglobin target status for the subsequent 3 months if the hemoglobin level met the criterion of 110–130 g/L at a given follow-up visit. Time to hemoglobin target non-attainment was defined as the cumulative duration (in months) from baseline until the hemoglobin level first fell outside the target range.

The standard deviation of residual hemoglobin (Res-SD) was used to assess hemoglobin variability (23).

Res-SD: Res-SD was defined as the square root of the sum of the squared differences between the observed hemoglobin values and the linear regression-predicted values, divided by the number of observations. The formula is as follows:


$$\mathrm{Res}-\mathrm{SD}=\sqrt{\frac{\sum_{i=1}^{n}{\left(Hb_{\mathrm{obs},i}-Hb_{\text{pred},i}\right)}^{2}}{n}}$$

### Treatment methods

In both patient groups, the starting dose of roxadustat was 100 mg three times per week for patients weighing 45–60 kg and 120 mg three times per week for patients weighing ≥ 60 kg. Dose-reduction group: The dose was adjusted using a predefined gradient (250 mg, 200 mg, 150 mg, 120 mg, 100 mg, 70 mg, 50 mg, 40 mg, and 20 mg) based on changes in hemoglobin levels to maintain the target range. Frequency-reduction group: The administration frequency was adjusted using a predefined gradient (3 times weekly, 2 times weekly, or 1 time weekly) based on changes in hemoglobin levels to maintain the target range. All patients were required to follow the dose-adjustment rule according to the hemoglobin response outlined in the study by Chen et al. (21). The specific adjustment regimen is detailed in Supplementary Table 1. Oral and intravenous iron supplementation was permitted, and blood transfusion therapy was allowed when hemoglobin levels were below 60 g/L.

### Statistics

Means ± SDs or medians (interquartile range) for continuous variables and proportions for categorical variables were calculated. Differences in baseline characteristics between the dose-reduction group and the frequency-reduction group were compared using *t*-tests, Kruskal–Wallis tests, or chi-squared tests, respectively. Comparisons of continuous measurements within each group were performed using repeated measures ANOVA. The association between tapering strategies and hemoglobin target non-attainment was evaluated using Cox proportional hazards models (hazards ratio [HR] and 95% confidence interval [95% CI]), without or with adjustments for age, sex, history of diabetes mellitus, PD vintage, mean daily roxadustat exposure and baseline BMI, arterial pressure, 24-h urine volume, weekly total Kt/V score, phosphorus, intact parathyroid hormone (iPTH), serum albumin, C-reactive protein, and ferritin. Moreover, we further divided the patients into subgroups based on sex (male vs. female), age (< 45 vs. ≥ 45 years), BMI (< 24 vs. ≥ 24 kg/m^2^), mean serum albumin level (< 35 vs. ≥ 35 g/L), mean serum ferritin level (< 200 vs. ≥ 200 ng/mL), history of diabetes mellitus (yes vs. no), PD vintage (< 14 vs. ≥ 14 month), and clinical center (clinical center 1, 2, 3). Potential effect modifiers in the relationship between tapering strategies and hemoglobin target non-attainment were evaluated using subgroup analysis, and their interactions were assessed.

A two-tailed *p*-value of < 0.05 was considered statistically significant in all the analyses. SPSS software package version 22.0 and R software were used for all data analyses.

## Results

### Participant characteristics

As shown in the flowchart (Figure 1), a total of 402 PD patients were included in the final analyses. Among them, the mean age was 45.3 ± 13.8 years, 55.5% were male, and the mean baseline SBP and DBP were 136.2 ± 17.2 and 87.1 ± 10.4 mmHg, respectively. The patients in the frequency-reduction group were more likely to have a longer PD vintage and a lower prevalence of diabetes. Detailed baseline characteristics are presented in Table 1.

**Table 1:** Demographic, clinical, and laboratory characteristics of the patients at baseline.

Characteristic	Total	Dose-reduction	Frequency-reduction	*p-*value
*N*	402	215	187	
Male, No. (%)	223 (55.5)	117 (54.4)	106 (56.7)	0.649
Age, y	45.3 ± 13.8	45.8 ± 13.1	44.7 ± 14.6	0.327
BMI	22.6 ± 3.9	22.3 ± 3.7	23.0 ± 4.1	0.216
Diabetes mellitus	64 (15.9)	43 (20.0)	21 (11.2)	**0.017**
SBP, mmHg	136.2 ± 17.2	136.6 ± 17.1	135.7 ± 14.6	0.222
DBP, mmHg	87.1 ± 10.4	87.2 ± 10.3	87.0 ± 10.4	0.668
Iron supplementation, No. (%)	203 (50.4)	111 (51.6)	92 (49.1)	0.119
Baseline laboratory examinations				
Phosphorus, mmol/L	1.6 ± 0.5	1.6 ± 0.5	1.6 ± 0.5	0.959
Blood hemoglobin, g/L	115.3 ± 5.6	116.2 ± 6.0	114.4 ± 5.1	0.133
Serum albumin, g/L	37.0 ± 6.1	36.4 ± 6.2	37.5 ± 5.9	0.138
Serum creatinine, μmol/L	925.6 ± 322.3	912.8 ± 346.2	940.2 ± 292.8	0.201
CRP, mg/L	4.3 ± 8.2	3.7 ± 5.8	5.0 ± 10.2	0.493
iPTH, pg/mL	421.8 ± 405.2	421.9 ± 431.3	421.8 ± 374.1	0.999
Ferritin, ng/mL	224.3 ± 228.0	211.9 ± 210.5	238.2 ± 246.0	0.409
Transferrin saturation, %	28.12 ± 16.16	28.07 ± 16.02	28.16 ± 16.32	0.960
Total cholesterol, mmol/L	4.0 (3.3, 4.9)	3.9 (3.2, 4.8)	4.0 (3.3, 5.0)	0.353
Baseline PD characteristics				
PD vintage, mo	28.6 ± 26.7	27.9 ± 27.8	29.4 ± 25.1	**<0.001**
24-h UF volume, ml/d	350.0 (200.0, 612.5)	425.0 (312.5, 637.5)	312.5 (200.0, 600.0)	0.342
24-h Urine volume, ml/d	600.0 (350.0, 900.0)	400.0 (212.5, 712.5)	600.0 (400.0, 900.0)	0.145
Dialysate GLUC, %	1.5 ± 0.1	1.5 ± 0.1	1.5 ± 0.1	0.222
Total Kt/V score	2.1 ± 0.2	2.1 ± 0.1	2.1 ± 0.2	0.548
Cause of ESRD				
Primary glomerular disease, No. (%)	232 (57.7)	122 (56.7)	110 (58.8)	
Hypertensive nephropathy, No. (%)	20 (4.9)	11 (5.11)	9 (4.8)	
Diabetic nephropathy, No. (%)	20 (4.9)	12 (5.5)	8 (4.2)	
Other, No. (%)	110 (27.3)	50 (23.2)	60 (32.0)	

Continuous variables are presented as mean ± SD or IQR (25th, 75th), while categorical variables are presented as No. (%). *p*-values were calculated to compare differences between the two groups using *t*-test, Kruskal–Wallis test, or chi-squared tests for continuous and categorical variables, respectively.

BMI, body mass index; SBP, systolic blood pressure; DBP, diastolic blood pressure; iPTH, intact parathyroid hormone; ESRD, end-stage renal disease; CRP, C-reactive protein; PD, peritoneal dialysis; UF, ultrafiltration.Bold values indicate statistical significance at *p* < 0.05.

### Mean hemoglobin levels and hemoglobin target attainment rates

**Figure 2:**
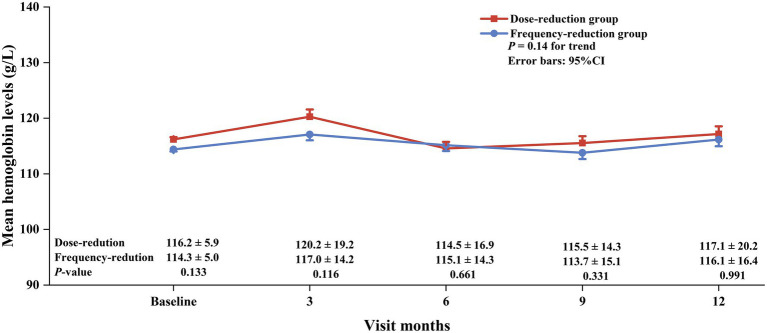
Mean hemoglobin levels in the dose-reduction and frequency-reduction groups during the follow-up period. *p* for trend compared the hemoglobin change trend.

**Figure 3:**
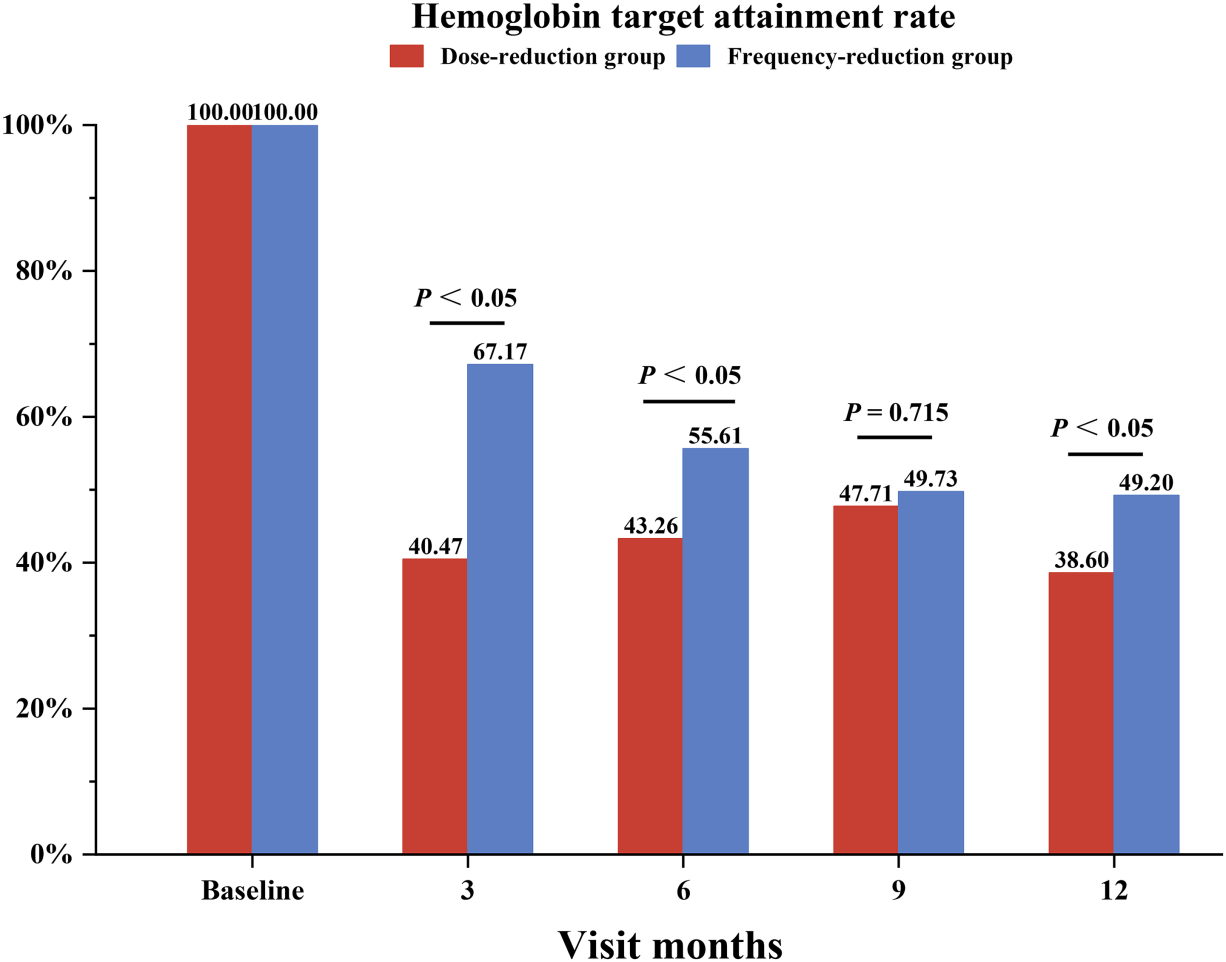
Hemoglobin target attainment rates in the dose-reduction and frequency-reduction groups at baseline and each follow-up visit. Target hemoglobin levels were 110–130 g/L.

We first analyzed the mean hemoglobin levels and hemoglobin target attainment rates during the follow-up period. As shown in Figures 2, 3, the mean hemoglobin levels were 116.2 ± 5.8, 120.2 ± 19.2, 114.5 ± 16.9, 115.5 ± 14.3, and 117.1 ± 20.2 in the dose-reduction group and 114.3 ± 5.0, 117.0 ± 14.2, 115.1 ± 14.3, 113.7 ± 15.1, and 116.1 ± 16.4 in the frequency-reduction group at baseline and at months 3, 6, 9, and 12, respectively. Overall, there was no difference in the changing trend of hemoglobin (*p* = 0.14) or in hemoglobin levels at each visit (all *p* > 0.05). The patients in the frequency-reduction group had significant higher hemoglobin target attainment rates at months 3, 6, and 12 compared to those in the dose-reduction group (64.1% vs. 40.4%, *p* < 0.05; 55.6% vs. 43.2%, *p* < 0.05; 49.2% vs. 36.6%, *p* < 0.05).

### Hemoglobin variability

**Figure 4:**
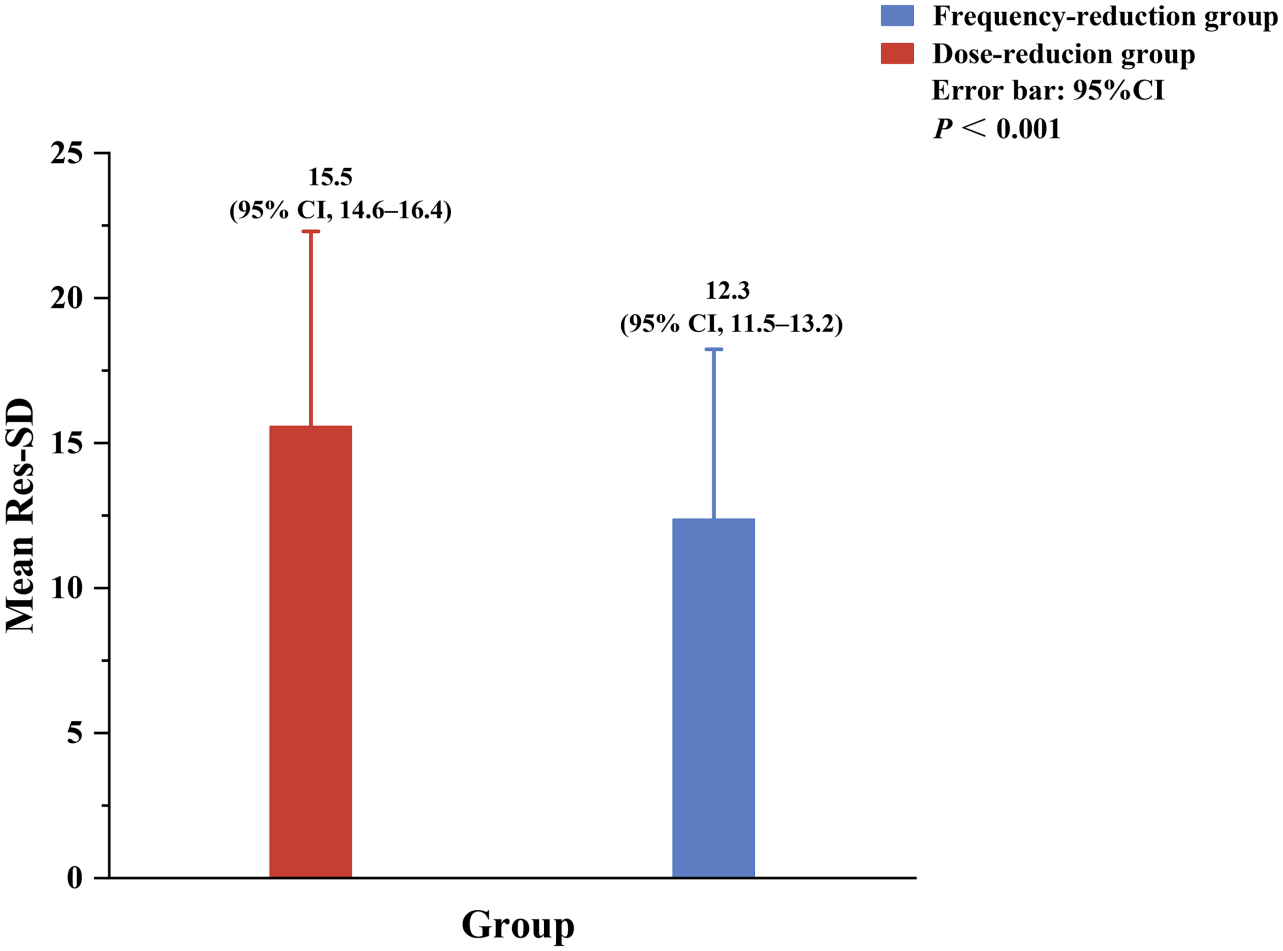
Res-SD scores in the dose-reduction and frequency-reduction groups. Res-SD was calculated by fitting a regression line to each patient’s hemoglobin measurements and then computing the square root of the sum of the squared vertical deviations between each actual hemoglobin value and its corresponding linear regression-predicted value. Res-SD, Standard deviation of residual hemoglobin.

We next calculated the Res-SD among the patients. The patients in the dose-reduction group had a mean Res-SD value of 15.5 (95% CI 14.6–16.4), which was significantly greater than that of the frequency-reduction group (12.3, 95% CI 11.5–13.2, *p* < 0.001; Figure 4).

### Association between tapering strategies and hemoglobin target non-attainment

We also analyzed the association between tapering strategies and hemoglobin target non-attainment using a Cox regression model. As shown in Table 2, the frequency-reduction group was associated with a lower risk of hemoglobin target non-attainment compared to the dose-reduction group, with an HR of 0.65 (95% CI 0.52–0.80, *p* < 0.001). After adjustment for age, sex, history of diabetes mellitus, PD vintage, mean daily roxadustat exposure and baseline BMI, arterial pressure, 24-h urine volume, weekly total Kt/V score, phosphorus, iPTH, serum albumin, C-reactive protein, and ferritin, the risk of hemoglobin target non-attainment remained lower in the frequency-reduction group, with a HR of 0.64 (95% CI 0.50–0.82, *p* < 0.001). A similar trend was found in the Kaplan–Meier curve using the log-rank test (Supplementary Figure 1).

**Table 2:** Association between hemoglobin target non-attainment and tapering strategies.

Hemoglobin target non-attainment, mo	Crude model^a^		Adjusted model^b^	
	HR (95% CI)	*p-*value	HR (95% CI)	*p-*value
Male	0.95 (0.77, 1.18)	0.664	1.02 (0.80, 1.30)	0.847
Age, y	1.00 (0.99, 1.00)	0.394	1.00 (0.99, 1.00)	0.270
BMI	0.99 (0.96, 1.02)	0.437	0.99 (0.96, 1.02)	0.538
Mean arterial pressure, mmHg	1.00 (0.99, 1.01)	0.519	0.99 (0.98, 1.01)	0.325
Diabetes mellitus	0.93 (0.69, 1.24)	0.603	0.86 (0.62, 1.18)	0.343
PD vintage, mo	1.00 (0.99, 1.00)	0.355	1.00 (0.99, 1.00)	0.749
Mean daily roxadustat exposure, mg/d	1.01 (1.00, 1.02)	0.142	1.00 (0.99, 1.01)	0.876
Phosphorus, mmol/L	1.06 (0.84, 1.33)	0.628	1.03 (0.80, 1.34)	0.797
iPTH, pg/mL	1.00 (1.00, 1.00)	0.929	1.00 (1.00, 1.00)	0.841
Serum albumin, g/L	1.00 (0.98, 1.01)	0.676	1.00 (0.98, 1.02)	0.661
CRP, mg/L	1.00 (0.99, 1.01)	0.993	1.01 (0.99, 1.02)	0.393
Serum ferritin, ng/mL	1.00 (1.00, 1.00)	0.681	1.00 (1.00, 1.00)	0.813
24-h UV, ml/d	1.00 (1.00, 1.00)	0.185	1.00 (1.00, 1.00)	0.670
Total Kt/V score	1.18 (0.42, 3.29)	0.757	1.04 (0.32, 3.26)	0.973
Groups				
Dose-reduction group	REF		REF	
Frequency-reduction group	0.65 (0.52, 0.80)	**< 0.001**	0.64 (0.50, 0.82)	**< 0.001**

BMI, body mass index; CRP, C-reactive protein; iPTH, intact parathyroid hormone; UV, urine volume.

a Crude model: No covariates were adjusted.

b Adjusted model: Adjusted for age, sex, history of diabetes mellitus, PD vintage, mean daily roxadustat exposure and baseline BMI, arterial pressure, 24-h urine volume, weekly total Kt/V score, phosphorus, iPTH, serum albumin, CRP, and ferritin.Bold values indicate statistical significance at *p* < 0.05.

### Stratified analyses

**Figure 5:**
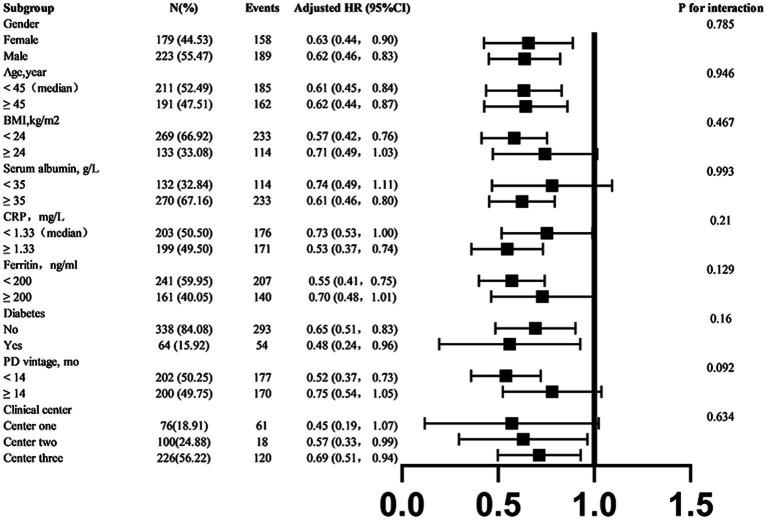
Subgroup analyses of the association between tapering strategies and hemoglobin target non-attainment. If a variable was not used for stratification, analyses were adjusted for age, sex, history of diabetes mellitus, PD vintage, clinical center, baseline arterial pressure, BMI, total Kt/V score, 24-h urine volume, serum albumin, CRP, and serum ferritin. BMI, body mass index; CRP, C-reactive protein.

To better understand other possible influencing factors in the relationship between tapering strategies and hemoglobin target non-attainment, we conducted additional exploratory subgroup analyses. None of the variables, including sex (male vs. female), age (< 45 vs. ≥ 45 years), BMI (< 24 vs. ≥ 24 kg/m^2^), mean serum albumin level (< 35 vs. ≥ 35 g/L), mean serum ferritin level (< 200 vs. ≥ 200 ng/mL), history of diabetes mellitus (yes vs. no), PD vintage (< 14 vs. ≥ 14 month), and clinical center (clinical center 1,2,3) significantly modified the association between tapering strategies and hemoglobin target non-attainment (all *p*-interactions > 0.05) (Figure 5).

## Discussion

In this multi-center retrospective study, the patients receiving the roxadustat frequency-reduction strategy exhibited higher hemoglobin target attainment rates, lower mean Res-SD values, and a reduced risk of hemoglobin target non-attainment compared to those in the dose-reduction group. These findings support frequency reduction as a potential tapering strategy for roxadustat in PD patients.

Roxadustat is increasingly used in clinical practice for anemia management in CKD patients, demonstrating benefits including enhanced iron metabolism, attenuated inflammation, reduced ESA resistance, oral administration convenience, and broad insurance coverage (16, 24–26). Within our center, adoption was significantly higher among PD patients, with approximately 70% receiving oral roxadustat compared to fewer than 20% continuing ESA injections. This disparity may be attributable to the standardized 1–3 month follow-up intervals for the PD population.

Current guidelines in China recommend initiating roxadustat at 100 mg (45–60 kg) or 120 mg (≥ 60 kg), administered thrice weekly (TIW) for dialysis-dependent patients. During the maintenance phase, target hemoglobin levels are maintained through stepwise dose reductions based on hemoglobin dynamics while retaining the TIW frequency (21). The rationale for this regimen is primarily based on pharmacokinetic studies: endogenous erythropoietin (EPO) peaks within 24 h post-dose and returns to baseline within 48 h after discontinuation, necessitating TIW dosing to sustain effective erythropoietic activity (27). However, the cascade of cellular signals drives the proliferation and differentiation of erythroid progenitor cells, leading to a stable lifespan of mature red blood cells and a sustained increase in hemoglobin levels (more than 60 days) (27). This provides a pharmacological basis for frequency reduction after anemia correction. A few trials have explored the feasibility of adopting a reduced fixed frequency of roxadustat after hemoglobin reached target levels (19, 20, 28). In a phase II study conducted in Japan in a non-dialysis-dependent population, patients were re-randomized into groups receiving roxadustat either TIW or once weekly after achieving target hemoglobin levels. No difference was observed in subsequent mean hemoglobin levels between the groups. Taken together, frequency reduction might be a potential tapering strategy in clinical practice.

In our study, nearly half of the patients (46.5%) adopted the frequency reduction strategy. Compared to the dose-reduction group, the patients in the frequency-reduction group exhibited higher hemoglobin target attainment rates at months 3, 6, and 12 and lower mean Res-SD values after calculation, along with similar mean hemoglobin levels and trends during subsequent follow-up visits. These results suggest the feasibility of the frequency reduction tapering strategy in peritoneal dialysis populations. Moreover, the frequency-reduction strategy seemed to be better for hemoglobin stability. Further analysis revealed that the dose-reduction group exhibited a higher mean roxadustat dose and a higher proportion of patients with hemoglobin > 130 g/L, while the proportions of patients with hemoglobin < 110 g/L were comparable at months 3, 6, 9, and 12 (Supplementary Figure 2). These findings suggest that relatively higher drug exposure might cause lower hemoglobin stability. Same protocol designs in non-dialysis CKD (29) and PD (30) populations also exhibited comparable efficacy, with significantly lower hemoglobin variability observed in patients receiving low-dose roxadustat compared to standard-dose therapy for anemia correction.

Roxadustat is commonly available in two tablet strengths: 20 mg and 50 mg. However, the 50 mg tablet was the only formulation stocked in many centers, which often precluded precise small dose reductions (e.g., from 100 mg to 70 mg). Consequently, physicians were sometimes compelled to make larger dose adjustments (e.g., from 100 mg to 50 mg). Due to concerns that such significant reductions could induce hemoglobin fluctuations, some clinicians preferred to reduce the administration frequency (e.g., from three times per week to twice per week) as a more manageable alternative. In addition, for patients with sub-optimal medication adherence or those seeking to reduce the administration frequency, physicians might prefer the frequency-reduction strategy to simplify the regimen. Furthermore, after achieving target hemoglobin levels, some physicians were hesitant to discontinue the drug completely due to concerns about potential hemoglobin decline, opting instead to reduce the dosing frequency to maintain a lower level of drug exposure. Therefore, both frequency- and dose-reduction strategies were applied in clinical practice. In our data, the patients in the frequency-reduction group exhibited higher hemoglobin target attainment rates and lower mean Res-SD values while maintaining similar mean hemoglobin levels and trends compared to the dose-reduction group. Therefore, in situations where the 20 mg tablet is unavailable or for patients who wish to reduce the administration frequency, the frequency-reduction strategy might be a potentially feasible alternative method. In other situations, both tapering strategies might be suitable.

Our study has several limitations that should be considered when interpreting the findings. First, as a retrospective cohort study, the design is inherently observational and cannot establish causal relationships between variables. Second, although we adjusted for a broad set of covariates in the analysis, residual confounding may persist due to unmeasured factors such as physicians’ individualized prescribing preferences, undocumented patient comorbidities, variations in medication adherence, and differences in iron dosage and administration patterns. Third, the frequency of hemoglobin monitoring in our study population may have been insufficient to capture more dynamic fluctuations, potentially affecting the accuracy of the conclusions. Fourth, the direct dose reduction of roxadustat from 100 mg to 50 mg, as observed in some of our clinical centers, may affect the validity of our conclusions. Finally, the study participants were recruited exclusively from southern China, which may limit the generalizability of the findings to other geographic or ethnic populations. Further validation in broader and more diverse cohorts is warranted.

## Conclusion

Collectively, our retrospective cohort study provides clinical evidence supporting the roxadustat frequency-reduction strategy for maintaining hemoglobin stability in PD patients, indicating its potential feasibility in this population. Further high-quality clinical studies are warranted to confirm these findings.

## Data Availability

The raw data supporting the conclusions of this article will be made available by the authors, without undue reservation.

## Supplementary material

SUPPLEMENTARY FIGURE 1 Kaplan-Meier survival analyses of the hemoglobin target non-attainment.

SUPPLEMENTARY FIGURE 2 Comparison of roxadustat dose between the dose-reduction group and the frequency-reduction group. *p* for trend compared roxadustat change trend.
