# Supplementary figures and images for: Frequency-reduction strategy of roxadustat in patients undergoing peritoneal dialysis: a multi-center retrospective cohort study
Source: Front Med (Lausanne). 2026 Jan 14;12:1708916. doi: 10.3389/fmed.2025.1708916 (PMC12847025; doi:10.3389/fmed.2025.1708916)

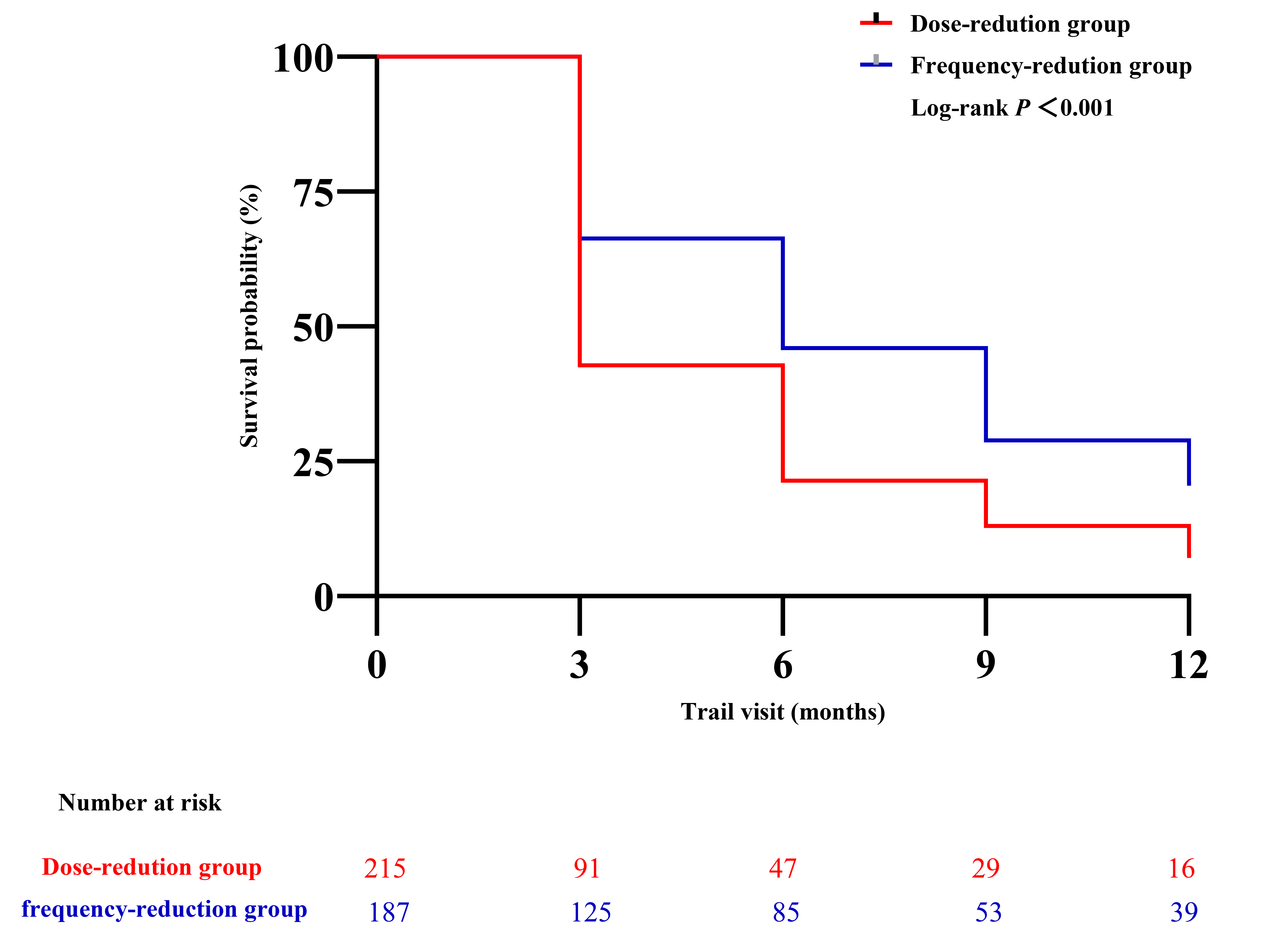

Supplement: SUPPLEMENTARY FIGURE 1 — Kaplan-Meier survival analyses of the hemoglobin target non-attainment. [file Image_1.tif]

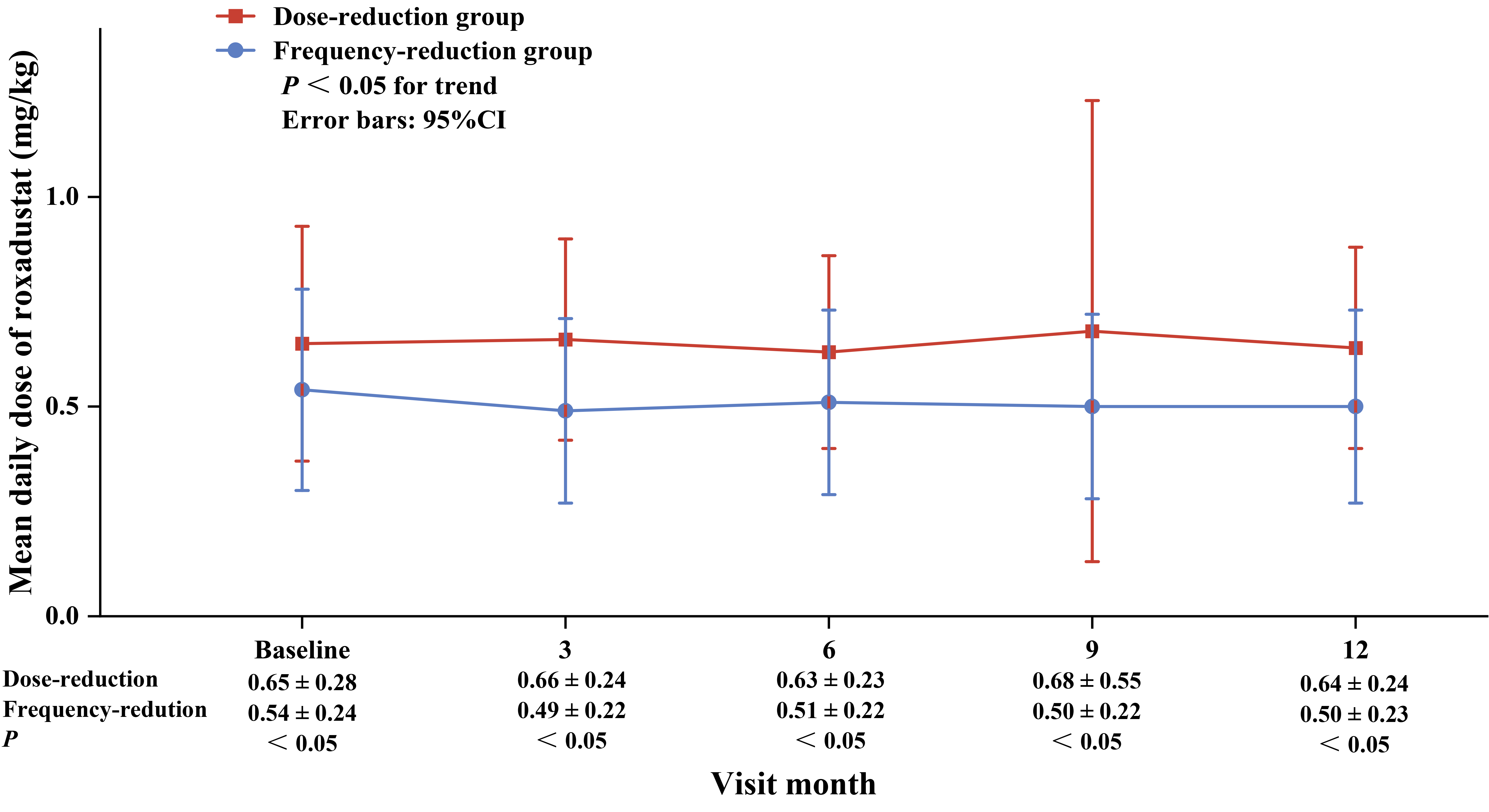

Supplement: SUPPLEMENTARY FIGURE 2 — Comparison of roxadustat dose between the dose-reduction group and the frequency-reduction group. p for trend compared roxadustat change trend. [file Image_2.tif]
