# Supplementary material for: Frequency-reduction strategy of roxadustat in patients undergoing peritoneal dialysis: a multi-center retrospective cohort study
Source: Front Med (Lausanne). 2026 Jan 14;12:1708916. doi: 10.3389/fmed.2025.1708916 (PMC12847025; doi:10.3389/fmed.2025.1708916)
Supplement: Supplementary file 3 [file Supplementary_file_1.docx]

**Supplemental Table 1.** Roxadustat dose adjustment algorithm

| Change in  hemoglobin  Over past 4 weeks (g/dL) | Hemoglobin level at dose-adjustment review visit (g/dl) | | | |
| --- | --- | --- | --- | --- |
|  | ＜ 10.5 | 10.5 to＜ 12.0 | ≥ 12.0 to＜ 13.0 | ≥ 13.0 |
| ＜ -1.0 | ↑ | ↑ | No change | Hold dosing , check hemoglobin, then resume dosing when hemoglobin <12.0 g/dL. |
| -1.0 to 1.0 | ↑ | No change | ↓ |  |
| ＞ 1.0 | No change | ↓ | ↓ |  |

Abbreviations: ↑ = increases; ↓ = decreases

Notes:

1. Dose-reduction group treatment:

 Dose increases (↑) and reductions (↓) were preset according to dose steps.

 The dose steps were as follows: 20, 40, 50, 70, 100, 120, 150, 200, and 250 mg.

For example, a dose increase at a dose of 70 mg resulted in 100 mg as the new dose. A dose reduction at a dose of 150 mg resulted in 120 mg as the new dose.

1. Frequency-reduction group treatment:

Frequency increases (↑) and reductions (↓) were preset as above.

The frequency steps were as follows: 1 times/week,2 times/week,3 times/week

For example, a frequency increase at a frequency of 1 times/week resulted in 2 times/week as the new frequency. A frequency reduction at a frequency of 3 times/week resulted in 2 times/week as the new frequency.

 The maximum dose was 2.5 mg/kg. The maximum frequency was 3 times/week. It was required to contact the Medical Monitor when dose or frequency adjustments were past the limits of the dose steps.

**Supplemental Table 2**. Number and proportion of patients with Hb at different levels in the two groups

| **Time point** | **Hb** | **Dose-reduction n (%)** | **Frequency-reduction n (%)** | ***P-*value^a^** |
| --- | --- | --- | --- | --- |
| Month 3 |  |  |  |  |
|  | ＜ 110 | 64 (29.8) | 43 (23.0) | 0.125 |
|  | ≥ 110 and ≤ 130 | 87 (40.5) | 120 (64.2) | **＜ 0.05** |
|  | ＞ 130 | 64 (29.8) | 24 (12.8) | **＜ 0.05** |
| Month 6 |  |  |  |  |
|  | ＜ 110 | 83 (38.6) | 64 (34.2) | 0.363 |
|  | ≥ 110 and ≤ 130 | 93 (43.3) | 104 (55.6) | **＜ 0.05** |
|  | ＞ 130 | 39 (18.1) | 21 (11.2) | **＜ 0.05** |
| Month 9 |  |  |  |  |
|  | ＜ 110 | 72 (33.5) | 71 (38.0) | 0.349 |
|  | ≥ 110 and ≤ 130 | 103 (47.9) | 93 (49.7) | 0.715 |
|  | ＞ 130 | 40 (19.1) | 23 (12.3) | **＜ 0.05** |
| Month 12 |  |  |  |  |
|  | ＜ 110 | 83 (38.6) | 65 (34.8) | 0.425 |
|  | ≥ 110 and ≤ 130 | 83 (38.6) | 92 (49.2) | **＜ 0.05** |
|  | ＞ 130 | 49 (22.8) | 30 (16.0) | **＜ 0.05** |

^a^At each time point, comparisons between groups were performed at each category of Hb level range by 2 × 2crosstabs.

Abbreviations: Hb, hemoglobin.
